# Red blood cell distribution width to albumin ratio predicts mortality in heart failure patients with pneumonia

**DOI:** 10.3389/fcvm.2026.1638901

**Published:** 2026-02-05

**Authors:** Hongyu Su, Mengzhao Yang, Haiyu Wang, Benchen Rao, Yuan Li, Zhengyu Chen, Zhigang Ren, Yuan Liu

**Affiliations:** 1 Department of Cardiology, The First Affiliated Hospital of Zhengzhou University, Zhengzhou, Henan, China; 2 Department of Infectious Diseases, The First Affiliated Hospital of Zhengzhou University, Zhengzhou, Henan, China; 3 Gene Hospital of Henan Province/Precision Medicine Center, The First Affiliated Hospital of Zhengzhou University, Zhengzhou, Henan, China; 4 Department of Cardiovascular Surgery, Fuwai Hospital, Chinese Academy of Medical Sciences & Peking Union Medical College/National Center for Cardiovascular Diseases, Beijing, China

**Keywords:** heart failure, machine learning, pneumonia, prognostic biomarker, red blood cell distribution width to albumin ratio

## Abstract

**Background:**

Heart failure (HF) patients with pneumonia admitted to the intensive care unit (ICU) face high mortality risks, yet current risk stratification tools lack precision. This study aims to evaluate the prognostic value of the red blood cell distribution width to albumin ratio (RAR) and develop a machine learning model for predicting 31-day in-hospital mortality in HF patients with pneumonia in ICU.

**Methods:**

We included the MIMIC-IV cohort and an external validation cohort from the First Affiliated Hospital of Zhengzhou University. The restricted cubic spline (RCS), Kaplan–Meier, and multivariable Cox regression analyses were used to estimate the RAR's prognostic value for 31-day in-hospital mortality. Boruta-selected features were used to build eight machine learning models.

**Results:**

In the Medical Information Mart for Intensive Care -IV (MIMIC-IV) cohort (*n* = 3,158), RCS revealed a linear relationship between RAR and all-cause mortality. Patients were subsequently stratified into high- and low-risk groups based on the median RAR value. Kaplan–Meier analysis showed higher mortality in patients with above-median RAR (*P* < 0.05), and each unit increase in RAR independently predicted a 13% higher mortality risk (HR 1.13, 95% CI 1.06–1.21; *P* < 0.001). Subgroup and sensitivity analyses confirmed RAR's prognostic consistency across demographic strata, comorbidities, and medication regimens. The LightGBM model, trained on Boruta-selected 13 optimal features, was identified as the best prediction model and demonstrated strong generalizability in internal (AUC = 0.735) and external validation cohort (*n* = 1,110 patients) (AUC = 0.733). SHapley Additive exPlanations analysis ranked RAR as the second most critical predictor in the model. A web-based tool (https://mengzhaoyang.shinyapps.io/PneumoHF-RAR_Predictor/) was developed for real-time risk assessment.

**Conclusions:**

This study identifies RAR as an effective prediction biomarker for HF patients with pneumonia and provides a clinical tool for precise risk profiling.

## 1 Introduction

Heart failure (HF), affecting over 64 million adults globally, carries heightened vulnerability to pneumonia—a critical complication that disproportionately impacts outcomes (1). Notably, HF patients exhibit pneumonia incidence rates exceeding general populations. The clinical consequences are dire: pneumonia independently amplifies mortality risk by 4.3-fold in HF with reduced ejection fraction and 3.8-fold in HF with preserved ejection fraction, with mortality peaking within 1 month post-infection (2). Alarmingly, 10% of first HF hospitalizations are pneumonia-triggered, making it the leading non-cardiovascular precipitant (3). These findings underscore an urgent need for early identification and risk stratification of HF patients with pneumonia. However, due to the complex interaction of various clinical factors, it is still a challenge to identify high-risk individuals accurately.

Red blood cell distribution width (RDW), a quantitative measure of erythrocyte volume heterogeneity, has shown prognostic value extending beyond its conventional role in anemia classification. In a *post hoc* analysis of the CHARM trial (Candesartan in Heart Failure: Assessment of Reduction in Mortality and Morbidity), after a median 34-month follow-up, each 1-standard deviation (SD) increase in RDW correlated with a 17% elevated risk of mortality among HF patients (4). The prognostic utility of RDW extends to diverse critical illnesses, with validated associations observed in respiratory failure (5), sepsis (6), and other conditions. Serum albumin is the predominant plasma protein, with its biological half-life spanning 19 days under physiological conditions (7). A study utilizing 1,070 cardiovascular disease patients from the National Health and Nutrition Examination Survey found a J-shaped relationship between serum albumin levels and long-term cardiovascular disease mortality. Lower albumin levels were significantly associated with a higher risk of mortality (8). A cohort study demonstrated that low serum albumin levels at admission were an independent predictor of long-term all-cause mortality in first-time acute myocardial infarction patients (9). The ratio of RDW to albumin (RAR) has emerged as a novel composite biomarker that integrates both inflammatory and nutritional aspects, potentially providing a more comprehensive prognostic assessment. A recent study demonstrated a strong association between RAR and all-cause as well as disease-specific mortality, with multiple cohort studies further validating its potential as a prognostic biomarker (10).

This study aims to investigate the association between RAR and 31-day in-hospital mortality in HF patients with pneumonia using real-world clinical data from the Medical Information Mart for Intensive Care -IV (MIMIC-IV) database. Furthermore, we seek to address the limitations of traditional methods by developing an interpretable machine learning model that dynamically incorporates RAR alongside multidimensional clinical features (including demographics, comorbidities, medications, and vital signs). The model underwent rigorous internal validation within the MIMIC-IV cohort, followed by external validation using an independent cohort from the First Affiliated Hospital of Zhengzhou University to ensure generalizability across diverse clinical settings. By leveraging machine learning's capacity to process complex data structures, our validated model aims to enhance mortality prediction accuracy, improve risk stratification, and facilitate early clinical decision-making. This approach may ultimately inform personalized treatment strategies for this high-risk population.

## 2 Methods

### 2.1 Data sources

This study employed a retrospective observational design, utilizing data extracted from the MIMIC-IV database, which contains detailed records of patients treated at Beth Israel Deaconess Medical Center from 2008 to 2019. (11) Adult patients with concurrent HF and pneumonia were identified to establish the primary study cohort for association analyses and prognostic modeling. The MIMIC-IV cohort was subsequently used for machine learning model development with internal validation. To further evaluate model generalizability, an independent cohort of patients with the same diagnoses was retrospectively enrolled from the First Affiliated Hospital of Zhengzhou University between January 2021 and December 2024.

### 2.2 Ethical statement

Ethical approval for the collection of the MIMIC-IV database was obtained by the original data-collecting institutions. As the MIMIC-IV database contains exclusively de-identified patient records, the analyses conducted in this study were exempt from additional ethics committee review and the requirement for informed consent. Author Hongyu Su obtained Collaborative Institutional Training Initiative (CITI) certification (ID: 68198847) prior to authorization for database access. External validation cohort utilization was approved by the Ethics Committee of the First Affiliated Hospital of Zhengzhou University, with a waiver for informed consent (Ethical approval number: 2025-KY-0267).

### 2.3 Inclusion and exclusion criteria

**Figure 1:**
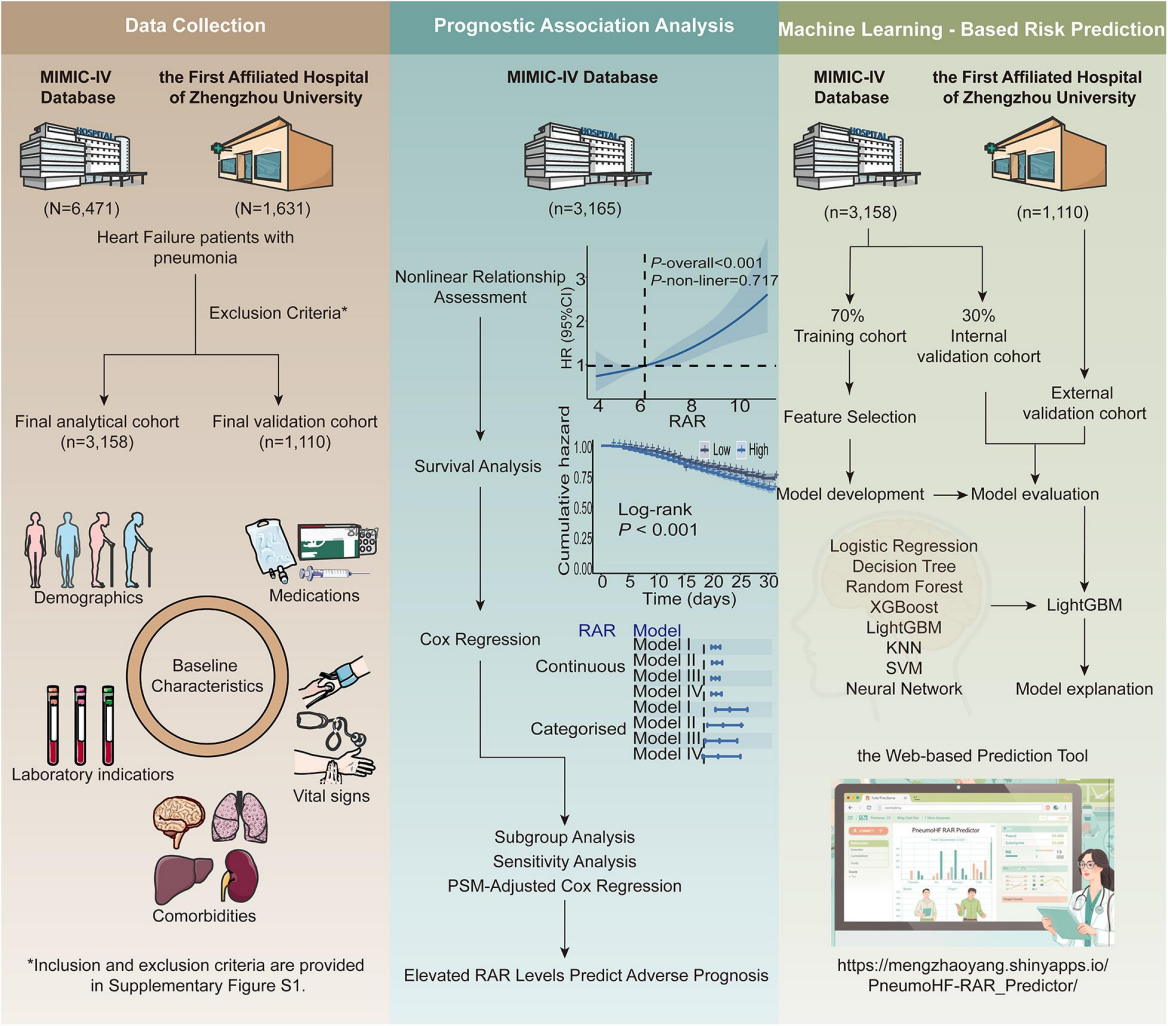
Overall design of the study.

For both the MIMIC-IV cohort and the external validation cohort, eligible patients were those who had concurrent diagnoses of HF and pneumonia recorded within the same hospitalization, as identified by the presence of corresponding International Classification of Diseases (ICD) codes (listed in Supplementary Table S1). Neither HF nor pneumonia was required to be the primary discharge diagnosis, and patients were included regardless of the diagnostic listing order. This inclusive definition was adopted to capture all critically ill patients affected by the coexistence of HF and pneumonia, reflecting real-world clinical practice in which diagnostic coding priority may vary according to presentation or administrative factors rather than true disease severity. Exclusion criteria included: (1) age <18 years, (2) the length of Intensive Care Unit (ICU) stay <24 h, and (3) absence of RDW and albumin results after ICU admission. The patient inclusion and exclusion process is shown in Supplementary Figure S1. The study flowchart and analytical framework are illustrated in Figure 1.

### 2.4 Data collection

Patient data were retrieved using PostgreSQL, comprising demographic variables [age, gender, and body mass index (BMI)], comorbidities (myocardial infarction, diabetes, hypertension, hepatopathy, atrial fibrillation, kidney disease, malignant tumor, chronic pulmonary disease, as well as cerebrovascular disease), along with medication use. Medications analyzed encompassed glucocorticoids, anticoagulants, beta blockers, renin-angiotensin-aldosterone system inhibitors (RAASi, including angiotensin-converting enzyme inhibitors and angiotensin receptor blockers), diuretics, antiplatelet drugs, angiotensin receptor-neprilysin inhibitors (ARNI), sodium-glucose cotransporter 2 inhibitors (SGLT2i), together with nonsteroidal anti-inflammatory drugs (NSAIDs). At the same time, key vital signs recorded for the first time after entering the ICU were extracted, including systolic blood pressure (SBP), diastolic blood pressure (DBP), heart rate (HR), respiratory rate (RR), and oxygen saturation (SpO2). In addition, hemodynamic and clinical monitoring parameters included central venous pressure (CVP), average left ventricular ejection fraction (LVEF), and cumulative fluid balance, defined as the net fluid input minus output during the first 24 h following ICU admission.

Disease characteristics were additionally collected. The diagnosis sequence was defined according to the relative ordering of ICD codes within the same hospitalization record. A value of 1 indicated that the ICD code for HF appeared before that for pneumonia, whereas a value of 0 indicated that the ICD code for pneumonia preceded that for HF. Ischemic HF was identified based on the presence of ICD codes corresponding to coronary artery disease, prior or acute myocardial infarction, or ischemic cardiomyopathy in the MIMIC-IV database. Patients without any of these diagnoses were categorized as having non-ischemic HF.

The initial laboratory data following ICU admission were collected, including red blood cell count (RBC), platelet count (Plt), white blood cell count (WBC), hemoglobin (Hb), RDW, neutrophil percentage (Neut), lymphocyte percentage (Lymph), absolute neutrophil count (Neut abs), absolute lymphocyte count (Lymph abs), monocyte count, alanine aminotransferase (ALT), aspartate aminotransferase (AST), alkaline phosphatase (ALP), albumin, creatinine (Cr), blood urea nitrogen (BUN), international normalized ratio (INR), prothrombin time (PT), partial thromboplastin time (PTT), potassium (K^+^), sodium (Na^+^), calcium (Ca^2+^), chloride (Cl^−^), phosphorus (P), bicarbonate (HCO₃⁻), glucose (Glu), troponin T (TnT), N-terminal pro–B-type natriuretic peptide (NTproBNP), C-reactive protein (CRP), creatine kinase-MB (CKMB), and creatine kinase (CK). For repeated measurements, only the first recorded values were included. The RAR was calculated according to the following formula:

$$\mathrm{RAR}=\frac{\mathrm{RDW}(\text{\%})}{\mathrm{Serum}\;\mathrm{albumin}\;(g/\mathrm{dL})}$$

The primary endpoint was all-cause in-hospital mortality. Follow-up initiated at admission and terminated upon the first occurrence of: (1) death during hospitalization, (2) discharge, or (3) 31-day observation completion.

### 2.5 Statistical analysis

Continuous variables were described as means with standard deviation (SD) or medians with interquartile ranges, depending on their distribution. Group comparisons were made using the Mann–Whitney *U*-test or Student's *t*-test for continuous variables and Pearson's chi-square test for categorical variables.

Variables with missing rates greater than 20% were excluded from the analysis. The excluded variables are explicitly indicated in Supplementary Table S2, which also presents the proportion of missing data for each variable. To handle the remaining missing data, multiple imputation was performed using the “mice” R package with predictive mean matching. Five imputed datasets were generated, and a fixed random seed was applied to ensure reproducibility. Outliers were identified and excluded using a *Z*-score–based approach, with values exceeding an absolute *Z*-score of 3 considered as outliers. The distributions of all imputed variables before and after imputation are presented in Supplementary Table S3, indicating that the imputation did not introduce substantial systematic bias.

Associations between RAR and mortality were modeled via restricted cubic splines (RCS). Subsequently, for visualization and group-based analyses, patients were dichotomized into high- and low-RAR groups based on the median RAR value, and Kaplan–Meier (KM) analysis was then performed to compare survival probabilities between these groups.

Subsequently, univariate and multivariable Cox proportional hazards models were applied to evaluate the association between continuous or categorized RAR and in-hospital mortality. Hazard ratios (HRs) with 95% confidence intervals (CIs) were reported. Four progressively adjusted models were constructed: Model I. Unadjusted; Model II. Adjusted for demographics (age, gender), vital signs (HR, RR, SpO2), and laboratory results (RBC, Plt, WBC, Hb, Neut, Lymph, ALT, AST, ALP, Cr, BUN, INR, PT, PTT, K^+^, Na^+^, Ca^2+^, Cl^−^, P, Glu, HCO3^−^); Model III. Adjusted for disease characteristics and comorbidities including diagnosis sequence, ischemic HF, myocardial infarction, diabetes, hypertension, hepatopathy, atrial fibrillation, kidney disease, malignant tumor, chronic pulmonary disease, cerebrovascular disease, as well as concomitant medication use (glucocorticoids, anticoagulant, beta blockers, RAASi, diuretic, antiplatelet drug, and NSAIDs); Model IV: Fully adjusted model incorporating all covariates from Model II and III. ARNI and SGLT2i were not included in the multivariable analyses due to extreme imbalance in the data, which could lead to unstable estimates.

Subgroup analyses stratified by age, gender, disease characteristics, comorbidities, and medication regimens evaluated RAR's prognostic value across patient subgroups. Sensitivity analyses assessed seven medication classes (glucocorticoids, anticoagulants, antiplatelet agents, beta blockers, diuretics, RAASi, NSAIDs) for outcome associations. To further address potential confounding, we performed propensity score matching (PSM) using multivariable logistic regression with covariates spanning demographics, vital signs, laboratory parameters, and comorbidities. A 1:1 nearest-neighbor matching (caliper = 0.15 SD of logit-transformed scores) balanced baseline characteristics while minimizing data exclusion. Covariate balance after matching was assessed using standardized mean differences (SMDs), with an SMD <0.10 considered indicative of adequate balance. Cox regression was subsequently applied to assess mortality risk associations with RAR in both continuous and dichotomized forms.

### 2.6 Machine learning analysis

The MIMIC-IV cohort was randomly partitioned into training (70%) and internal validation (30%) sets. Baseline characteristics between the training and internal validation cohorts were compared to confirm acceptable balance (Supplementary Table S5). In addition, baseline characteristics of the overall MIMIC-IV cohort and the external validation cohort were compared to assess cohort comparability and external generalizability (Supplementary Table S6). A Boruta algorithm-driven feature selection process was implemented in the training set, iteratively comparing predictor importance against permuted shadow features to retain statistically significant variables. Collinearity among selected variables was subsequently assessed, and highly correlated features were removed. Continuous variables were standardized using *z*-score normalization prior to model training. Eight machine learning models—Logistic Regression, Decision Tree, Random Forest, k-nearest neighbor (KNN), Support Vector Machine (SVM), Neural Network, eXtreme Gradient Boosting (XGBoost), and Light Gradient Boosting Machine (LightGBM)—were constructed on the training cohort. Hyperparameter tuning for LightGBM was conducted using 5-fold cross-validation within the training set, incorporating L1/L2 regularization and early stopping to mitigate overfitting. Hyperparameter tuning strategies for the remaining models followed similar cross-validation principles, with model-specific parameter settings detailed in the accompanying analysis code. The performance of all machine learning models was evaluated on the internal validation cohort and external validation cohort. Receiver operating characteristic (ROC) curves and decision curve analysis (DCA) were generated, with the area under the curve (AUC) serving as the primary metric for model comparison. To statistically compare AUCs in the external validation cohort, we performed pairwise DeLong's tests and applied Holm correction for multiple comparisons. In addition to discrimination, we assessed overall performance and calibration in the external validation cohort using the Brier score, calibration intercept and slope, and the Hosmer–Lemeshow test with 10 groups. To evaluate the incremental predictive value of RAR within the final predictive model, we performed a formal comparison between the optimal LightGBM model including RAR and an identical model excluding RAR (reduced model). Incremental performance was assessed using the area under the receiver AUC, integrated discrimination improvement (IDI), and net reclassification index (NRI) in both the internal and external validation cohorts. AUC was used as the primary performance metric, while IDI and NRI were used to assess incremental improvements in risk discrimination and reclassification. The optimal model underwent interpretability analysis using SHapley Additive exPlanations (SHAP) analysis to quantify feature contributions at both global and individual levels. To facilitate clinical translation, a web-based platform integrating the final model was deployed via R Shiny, enabling real-time mortality risk stratification.

Data analyses were conducted using R (v4.5.2), with statistical significance defined as a two-tailed *P* < 0.05.

## 3 Results

### 3.1 Baseline characteristics

We initially enrolled 4,618 patients from the MIMIC-IV database. After excluding patients with abnormal values, a total of 3,158 patients were included in the final analysis, comprising 2,586 survivors and 572 non-survivors. Table 1 presents the baseline characteristics of the survivor and non-survivor groups. Compared to non-survivors, survivors were markedly younger. Regarding comorbidities, the prevalence of hypertension was substantially higher in survivors, whereas non-survivors had considerably higher rates of hepatopathy, kidney disease, malignant tumor, and cerebrovascular disease. In terms of medication use, survivors showed greater utilization of glucocorticoids, beta blockers, RAASi, diuretics, antiplatelet drugs, and NSAIDs. Compared to survivors, non-survivors exhibited elevated WBC, neutrophil percentage, Cr, BUN, INR, PT, PTT, and phosphorus, alongside reduced Plt, lymphocyte percentage, and HCO₃⁻.

**Table 1:** Baseline characteristics of the survivor and non-survivor groups.

Variable	Overall	Survivor	Non-survivor	*P*-value
	(*n* = 3,158)	(*n* = 2,586)	(*n* = 572)	
RAR	5.34 ± 1.41	5.22 ± 1.34	5.88 ± 1.62	<0.001
Demographics				
Male, *n* (%)	1,843 (58.4%)	1,500 (58.0%)	343 (60.0%)	0.416
Age, years	73.22 ± 12.70	72.68 ± 12.88	75.66 ± 11.55	<0.001
Comorbidities, *n* (%)				
Myocardial infarction	962 (30.5%)	779 (30.1%)	183 (32.0%)	0.407
Diabetes	1,359 (43.0%)	1,128 (43.6%)	231 (40.4%)	0.172
Hypertension	2,505 (79.3%)	2,071 (80.1%)	434 (75.9%)	0.028
Hepatopathy	384 (12.2%)	287 (11.1%)	97 (17.0%)	<0.001
Atrial fibrillation	1,183 (37.5%)	954 (36.9%)	229 (40.0%)	0.174
Kidney disease	1,334 (42.2%)	1,066 (41.2%)	268 (46.9%)	0.015
Malignant tumor	396 (12.5%)	307 (11.9%)	89 (15.6%)	0.019
Chronic pulmonary disease	1,377 (43.6%)	1,131 (43.7%)	246 (43.0%)	0.786
Cerebrovascular disease	518 (16.4%)	401 (15.5%)	117 (20.5%)	0.005
Disease characteristics, *n* (%)				
Diagnosis Sequence = 1	1,061 (33.6%)	892 (34.5%)	169 (29.5%)	0.027
Ischemic HF	1,748 (55.4%)	1,412 (54.6%)	336 (58.7%)	0.079
Medications, *n* (%)				
Glucocorticoid	1,408 (44.6%)	1,129 (43.7%)	279 (48.8%)	0.029
Anticoagulant	2,679 (84.8%)	2,184 (84.5%)	495 (86.5%)	0.233
Beta blocker	2,487 (78.8%)	2,119 (81.9%)	368 (64.3%)	<0.001
RAASi	1,176 (37.2%)	1,085 (42.0%)	91 (15.9%)	<0.001
Diuretic	2,824 (89.4%)	2,336 (90.3%)	488 (85.3%)	0.001
Antiplatelet drug	2,095 (66.3%)	1,760 (68.1%)	335 (58.6%)	<0.001
NSAIDs	2,101 66.5%)	1,771 (68.5%)	330 (57.7%)	<0.001
First laboratory results upon ICU admission				
RBC, m/ul	3.51 ± 0.77	3.53 ± 0.77	3.46 ± 0.78	0.06
Plt, K/ul	213.21 ± 97.87	216.62 ± 97.61	197.77 ± 97.61	<0.001
WBC, K/ul	12.60 ± 6.59	12.48 ± 6.53	13.15 ± 6.86	0.028
Hb, g/dl	10.30 ± 2.19	10.33 ± 2.20	10.16 ± 2.15	0.083
Neut, %	79.70 ± 10.48	79.35 ± 10.51	81.28 ± 10.24	<0.001
Lymp, %	10.67 ± 7.42	11.07 ± 7.48	8.86 ± 6.87	<0.001
ALT, IU/L	53.88 ± 116.60	51.59 ± 113.72	64.26 ± 128.41	0.019
AST, IU/L	76.58 ± 159.26	71.71 ± 150.91	98.59 ± 191.16	<0.001
ALP, IU/L	103.65 ± 61.72	102.85 ± 61.53	107.28 ± 62.51	0.12
Cr, mg/dl	1.67 ± 1.20	1.63 ± 1.18	1.85 ± 1.27	<0.001
BUN, mg/dl	35.38 ± 22.04	34.17 ± 21.59	40.84 ± 23.23	<0.001
INR	1.59 ± 0.73	1.57 ± 0.73	1.65 ± 0.74	0.035
PT, sec	17.25 ± 7.51	17.12 ± 7.45	17.86 ± 7.75	0.031
PTT, sec	36.17 ± 14.83	35.92 ± 14.66	37.29 ± 15.53	0.047
K^+^, mEq/L	4.27 ± 0.71	4.27 ± 0.71	4.30 ± 0.74	0.291
Na^+^, mEq/L	138.20 ± 5.17	138.20 ± 5.12	138.16 ± 5.42	0.855
Ca^2+^, mg/dl	8.41 ± 0.74	8.42 ± 0.74	8.38 ± 0.77	0.257
Cl^−^, mEq/L	101.86 ± 6.46	101.93 ± 6.40	101.51 ± 6.71	0.153
P, mg/dl	3.86 ± 1.23	3.80 ± 1.20	4.15 ± 1.32	<0.001
HCO3^−^, mEq/L	24.24 ± 5.49	24.36 ± 5.44	23.70 ± 5.68	0.009
Glu, mg/dl	152.80 ± 65.42	152.75 ± 65.78	153.01 ± 63.83	0.932
Vital signs				
RR, insp/min	21.02 ± 6.05	20.99 ± 6.02	21.13 ± 6.21	0.616
HR, bpm	91.35 ± 25.77	91.10 ± 25.50	92.46 ± 26.98	0.253
SpO2, %	95.78 ± 4.56	95.83 ± 4.45	95.51 ± 5.03	0.129

RAR, red blood cell distribution width to albumin ratio; HF, heart failure; RAASi, renin-angiotensin-aldosterone system inhibitors; NSAIDs, nonsteroidal anti-inflammatory drugs; RAASi, RBC, red blood cell count; Plt, platelet count; WBC, white blood cell count; Hb, hemoglobin; Neut, neutrophil percentage; Lymph, lymphocyte percentage; ALT, alanine aminotransferase; AST, aspartate aminotransferase; ALP, alkaline phosphatase; Cr, creatinine; BUN, blood urea nitrogen; INR, international normalized ratio; PT, prothrombin time; PTT, partial thromboplastin time; K^+^, potassium; Na^+^,sodium; Ca^2+^, calcium; Cl^−^,chloride; P, phosphorus; HCO₃⁻, bicarbonate; Glu, glucose; RR, respiratory rate; HR, heart rate; SpO2, Oxygen saturation. Diagnosis Sequence = 1: HF diagnosis precedes pneumonia.

### 3.2 Relationship between RAR and in-hospital mortality in HF patients with pneumonia

**Figure 2:**
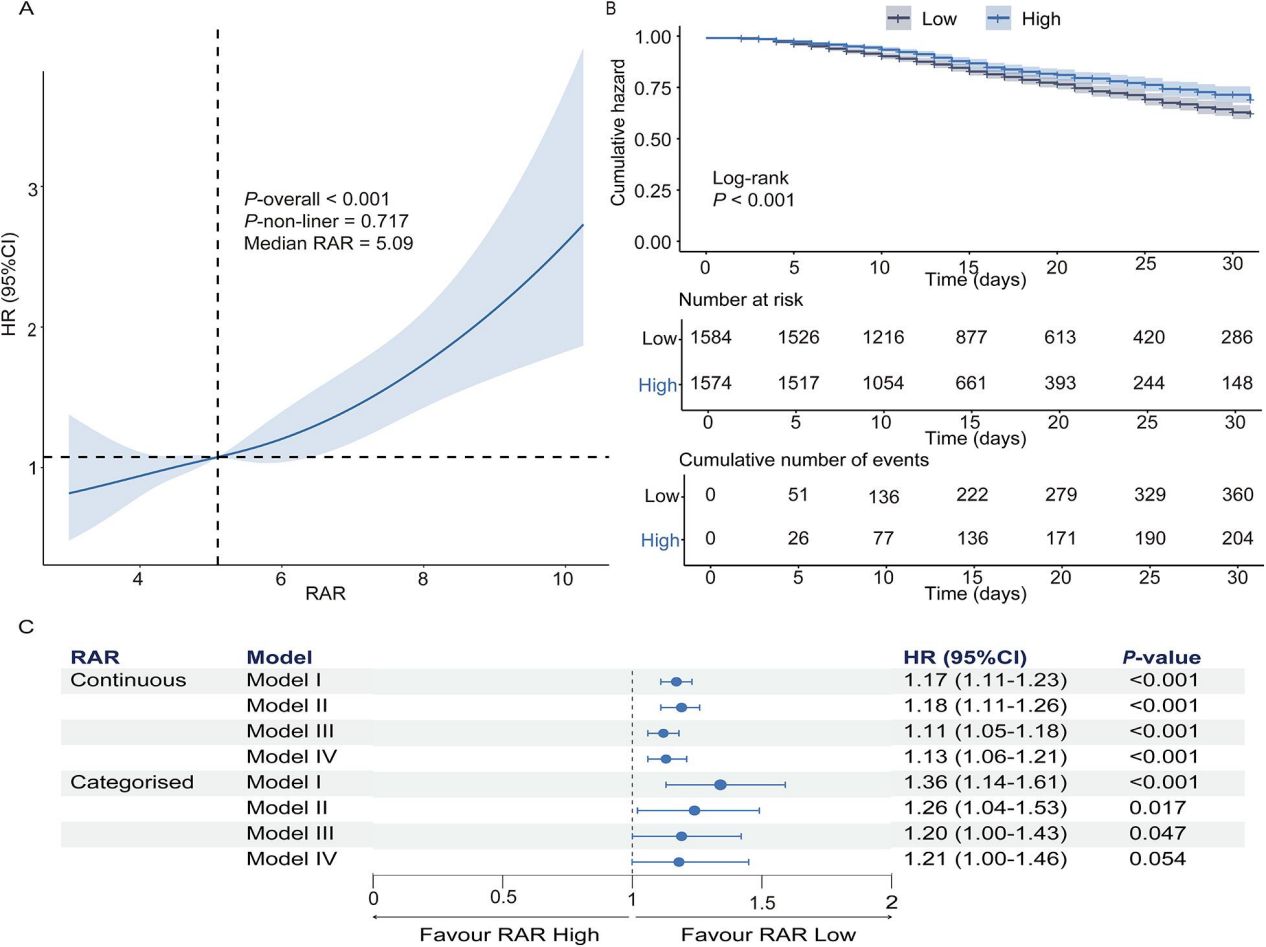
The prognostic value of RAR in predicting 31-day in-hospital mortality in heart failure patients with pneumonia. **(A)** Restricted cubic spline analysis demonstrates the relationship between RAR and hazard ratio for 31-day in-hospital mortality. **(B)** Kaplan–Meier survival curves for the high-RAR and low-RAR groups. **(C)** Cox regression analysis assessing the association of continuous and categorized RAR with in-hospital mortality. Model I: Unadjusted; Model II: Adjusted for demographics, vital signs, and laboratory results; model III: adjusted for disease characteristics, medications, and comorbidities; model IV: fully adjusted model incorporating all covariates from models II and III. RAR, red blood cell distribution width-to-albumin ratio.

RCS regression analysis revealed a significant linear association between RAR and in-hospital mortality in HF patients with concomitant pneumonia (overall *P* < 0.001; nonlinearity *P* = 0.717). For visualization and comparative analysis, patients were categorized into high- and low-RAR groups based on the median RAR value (5.09, Figure 2A). KM analysis demonstrated markedly higher 31-day all-cause mortality in the high-RAR group (log-rank *P* < 0.001, Figure 2B).

Multivariable Cox regression revealed that elevated RAR independently predicted in-hospital mortality after full adjustment (demographics, vital signs, laboratory parameters, comorbidities). As a continuous variable, each unit elevation in RAR conferred a 13% higher mortality risk [HR: 1.13 (95% CI: 1.06–1.21); *P* < 0.001]. When RAR was analyzed as a categorical variable, the high RAR group demonstrated a 1.28-fold mortality hazard relative to the low RAR group [HR: 1.21 (95% CI: 1.00–1.46); *P* = 0.054] (Figure 2C).

### 3.3 Subgroup analysis and sensitivity analysis

Subgroup analyses revealed no significant interaction effects across demographic strata, comorbid conditions, medication regimens, or diagnosis sequence (whether heart failure diagnosis preceded pneumonia or vice versa), and no interaction between ischemic and non-ischemic HF (Supplementary Figure S2). In medication-adjusted sensitivity analyses, continuous RAR maintained a robust mortality association across all models (Supplementary Figure S3).

Following PSM to mitigate confounding, 998 matched pairs of patients were generated. Covariate balance between the low- and high-RAR groups was substantially improved after matching, with all standardized mean differences below 0.10 (Supplementary Table S4 and Supplementary Figure S4). In the matched cohort, each 1-unit increase in RAR remained associated with a 14% higher risk of all-cause mortality [HR: 1.14, 95% CI: (1.05–1.25); *P* = 0.002] (Supplementary Figure S5).

### 3.4 Machine learning analysis

#### 3.4.1 Construction, validation, and evaluation of predictive models

**Figure 3:**
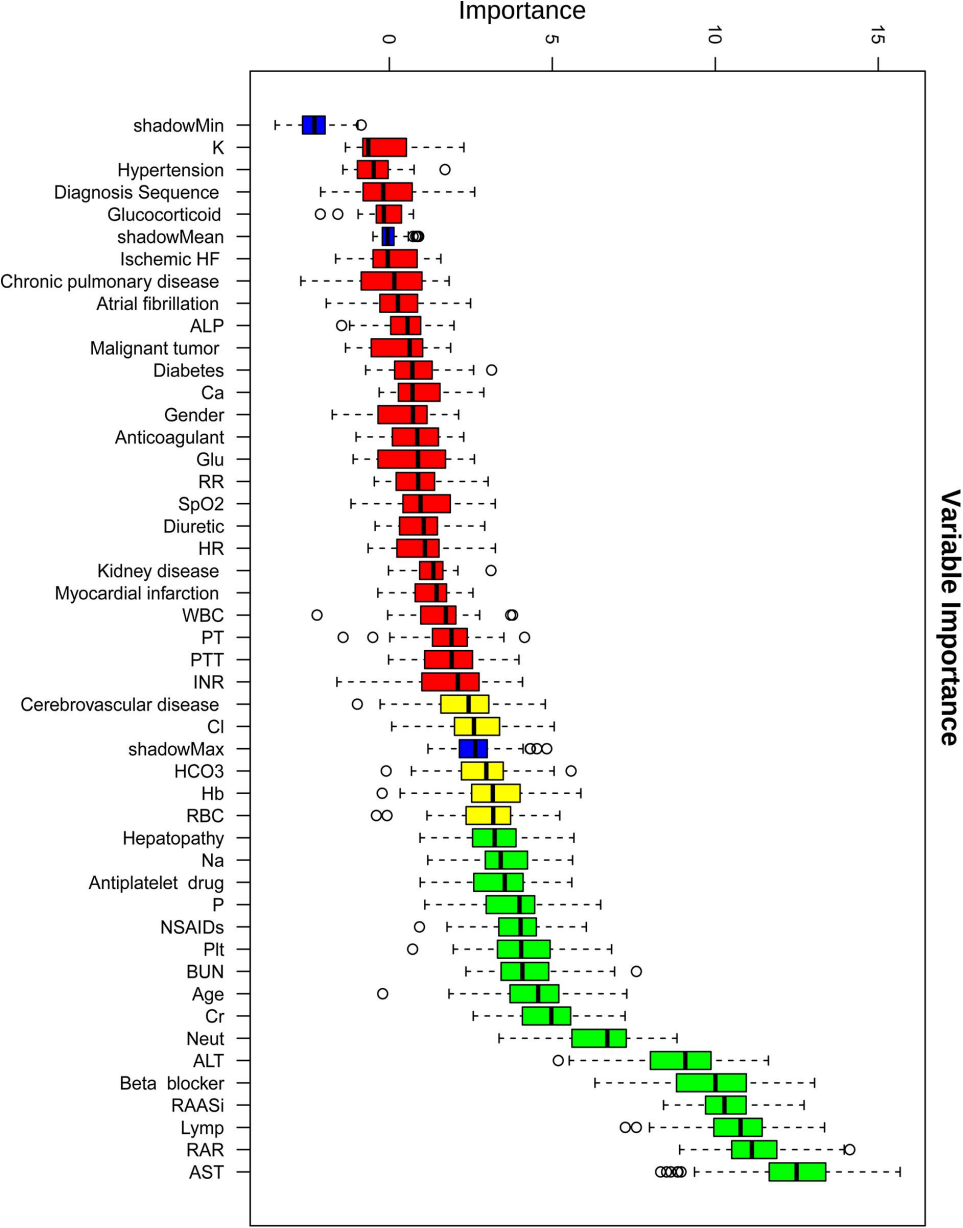
Variable importance ranking based on the boruta feature selection algorithm for predicting in-hospital mortality. This figure shows the variable importance scores of clinical features identified using the Boruta algorithm for predicting in-hospital mortality in heart failure patients with pneumonia. Boxplots represent the distribution of importance scores for each feature, with red indicating lower importance and green indicating higher importance.

Boruta algorithm-driven feature selection identified RAR as the second predictor of in-hospital mortality (Figure 3), following only serum AST levels. To reduce the impact of collinearity and enhance model stability, a collinearity analysis was performed. Based on collinearity, Boruta importance, and clinical relevance, the following variables were removed: antiplatelet drugs, ALT, and neut (Supplementary Figure S6).

**Figure 4:**
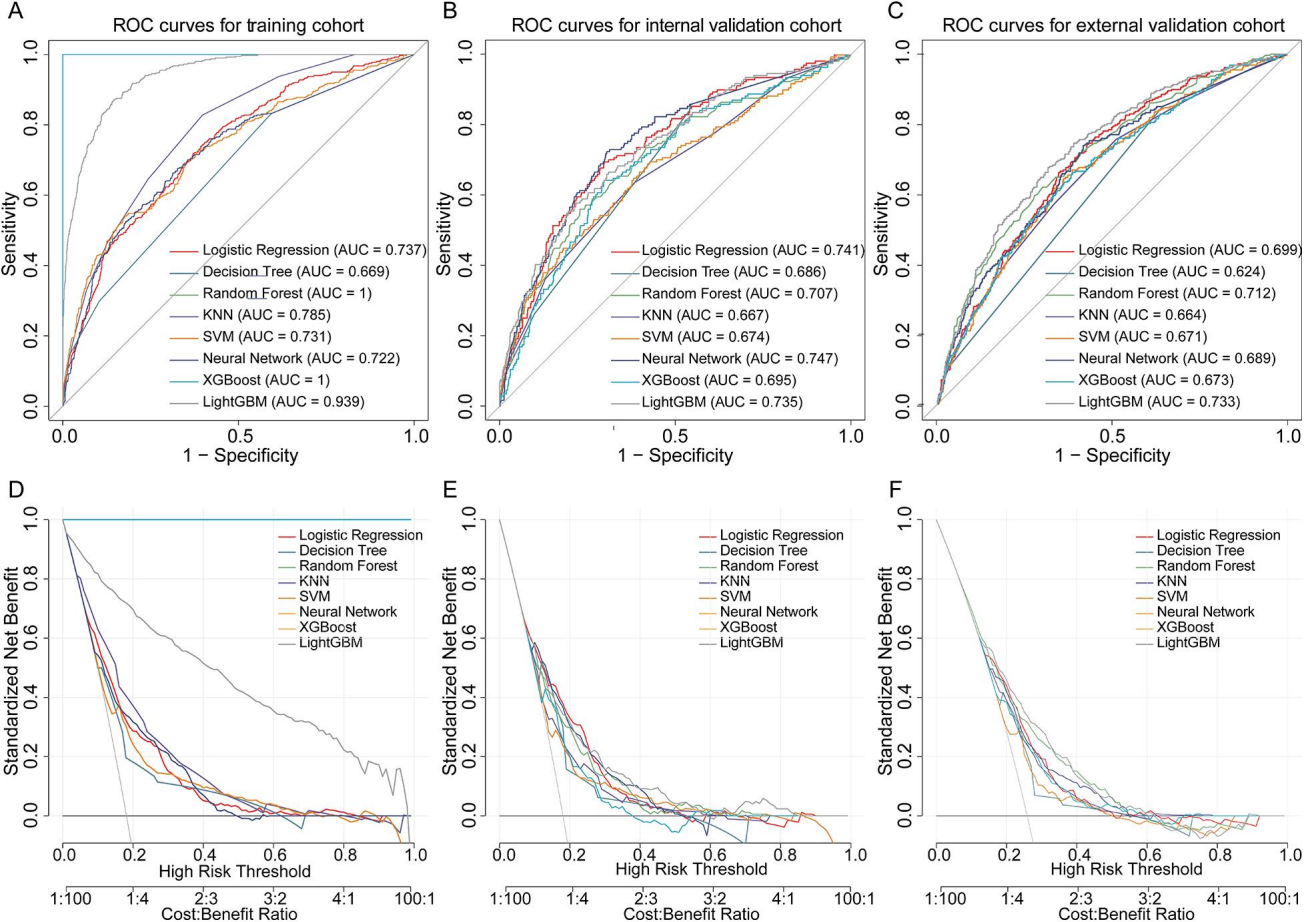
Performance of machine learning models in predicting in-hospital mortality in heart failure patients with pneumonia. **(A–C)** ROC curves of eight machine learning models in the training, internal validation, and external validation cohorts. **(D–F)** DCA curves for each cohort, demonstrating the net benefit of models across risk thresholds. ROC, receiver operating characteristic; DCA, decision curve analysis.

We developed eight machine learning models—Logistic Regression, Decision Tree, Random Forest, KNN, SVM, Neural Network, XGBoost, and LightGBM—to predict in-hospital mortality in HF patients with pneumonia. After hyperparameter tuning, all models were evaluated on internal and external validation cohorts. As presented in Figure 4, the LightGBM model achieved AUC values of 0.939, 0.735, and 0.733 in training, internal, and external validation cohorts, respectively and yielded the highest AUC in the external validation cohort. The close agreement between internal and external validation AUCs suggests acceptable generalizability across independent datasets. Notably, model selection was based on validation performance, with primary emphasis on external validation AUC. Pairwise DeLong tests with Holm correction in the external validation cohort indicated that the AUC difference between LightGBM and Random Forest was modest and not statistically significant (Supplementary Table S7), suggesting comparable discrimination between these top-performing models. In addition to discrimination, we reported Brier scores and calibration indices for all models in external validation to provide a comprehensive performance assessment (Supplementary Table S8). DCA further supported the potential clinical utility across clinically relevant risk thresholds (Figure 4).

To evaluate the incremental predictive value of RAR, we compared the full model (LightGBM with RAR) to a reduced model (excluding RAR) using AUC, IDI, and NRI in both the internal and external validation cohorts. In the internal validation cohort, the inclusion of RAR did not significantly improve the AUC (0.939 vs. 0.936, *Δ*AUC = 0.002, *P* = 0.53). However, RAR significantly improved risk discrimination, as indicated by an IDI of 0.022 (95% CI: 0.011–0.033, *P* < 0.001) and a continuous NRI of 0.189 (95% CI: 0.081–0.297, *P* < 0.001).

In the external validation cohort, the AUC improvement remained non-significant (0.735 vs. 0.720, ΔAUC = 0.015, *P* = 0.127), but the IDI was statistically significant (0.020, 95% CI: 0.003–0.038, *P* = 0.026), indicating consistent improvement in discrimination. The continuous NRI showed a positive trend (0.121) but did not reach statistical significance. Category-based NRI, using a predefined risk threshold of 0.20, did not demonstrate meaningful reclassification in either cohort, suggesting that the incremental value of RAR is primarily reflected in improved discrimination rather than categorical risk reassignment. Although the AUC improvement was not statistically significant in either cohort, AUC is less sensitive to improvements in well-performing models. In contrast, IDI and NRI are more sensitive to changes in risk discrimination, which explains the observed significant IDI and favorable NRI results.

#### 3.4.2 Model interpretability and practicality

**Figure 5:**
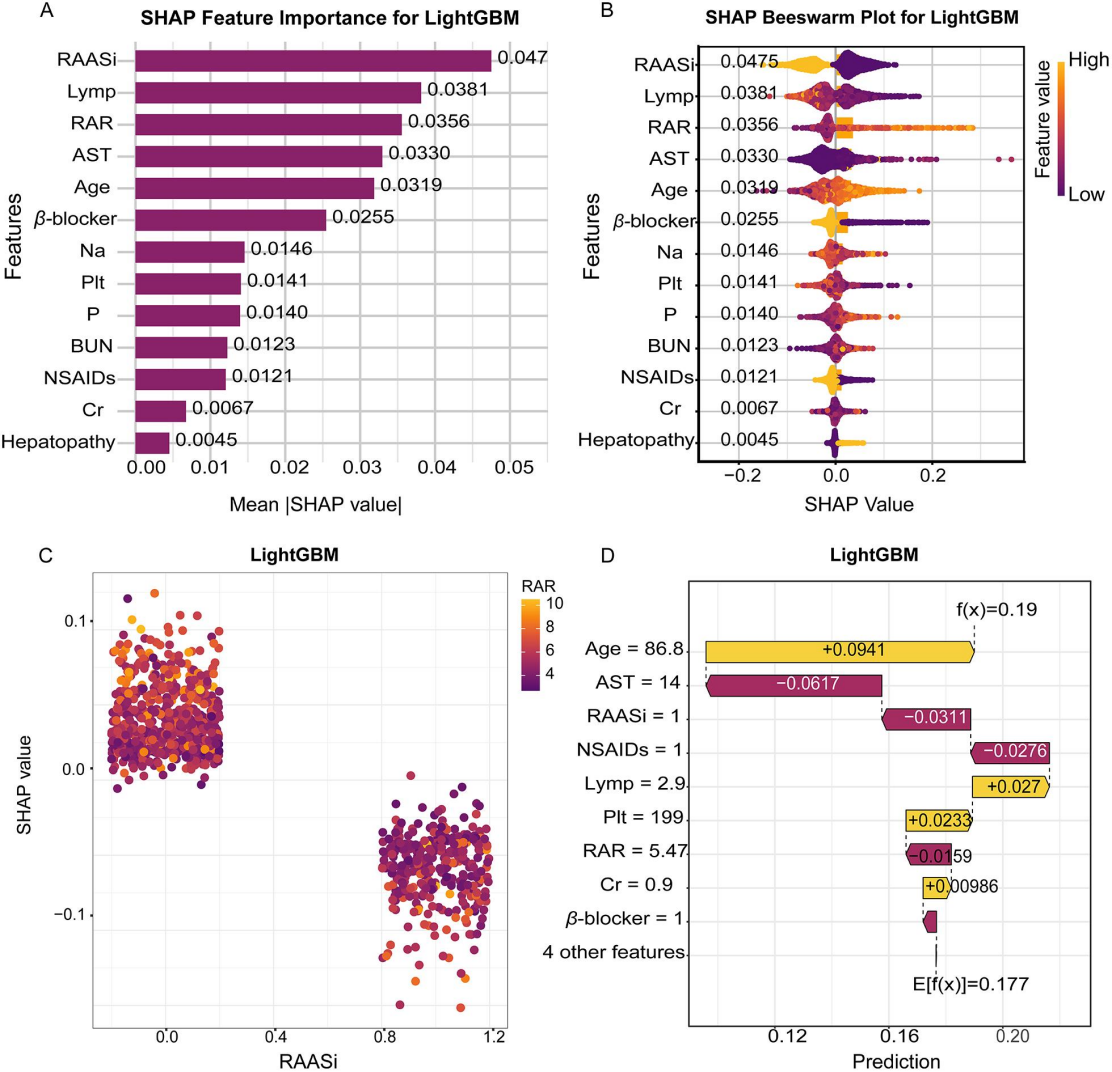
Interpretable SHAP analysis of the LightGBM model for in-hospital mortality prediction. **(A)** SHAP feature importance plot showing the mean absolute SHAP values for the top predictive variables. **(B)** SHAP beeswarm plot displaying the distribution of SHAP values for each feature across all samples. **(C)** SHAP dependence plot visualizing the relationship between RAASi and SHAP values. **(D)** Waterfall plot presenting the impact of key features on an individual prediction. SHAP, SHapley additive explanations; LightGBM, light gradient boosting machine; RAASi, renin-angiotensin-aldosterone system inhibitors.

To enhance model transparency, we applied SHAP values to interpret the final LightGBM model. Figures 5A,B present global SHAP feature importance and beeswarm plots, highlighting the most influential predictors of mortality risk. The analysis revealed that RAASi, lymphocyte percentage, and RAR were the top contributors to mortality prediction. Among these, RAASi use and higher lymphocyte percentage consistently showed negative SHAP values, indicating that these features are associated with a lower predicted mortality risk. In contrast, higher RAR values exhibited positive SHAP values, reflecting an increased mortality risk across the cohort. The impact of other variables incorporated into the model, such as age, AST, and beta blocker use, followed a similar pattern at the global level, with their respective SHAP values indicating their direction of influence on mortality risk.

Figures 5C,D provide further clarity at the individual level, where SHAP waterfall and decision plots illustrate how specific feature values contribute to the predicted mortality risk. In these plots, we observe that combinations of advanced age, elevated RAR, and the absence of guideline-directed therapies (e.g., RAASi and beta blockers) resulted in a higher predicted risk for certain patients. For example, older patients with elevated RAR and without RAASi treatment had significantly higher mortality risk predictions. These individual-level insights, driven by the additive contributions of each feature, provide clinicians with interpretable, transparent reasoning for the model's predictions, supporting evidence-based decision-making.

To facilitate the clinical translation of our machine learning model, we developed an interactive, web-based platform named PneumoHF - RAR - Predictor. This platform incorporates the optimized LightGBM model and is accessible at https://mengzhaoyang.shinyapps.io/PneumoHF-RAR_Predictor/. The user-friendly interface enables clinicians to input 13 key clinical variables, including demographics, comorbidities, laboratory values, and treatments, for real-time mortality risk prediction in HF patients with pneumonia.

## 4 Discussion

This study aimed to explore the prognostic value of RAR in predicting 31-day in-hospital mortality in HF patients with pneumonia. Our results demonstrate that RAR is a significant and reliable biomarker that can be integrated into machine learning models to improve mortality risk stratification in this high-risk population. This finding is supported by both traditional statistical methods and advanced machine learning techniques, highlighting the potential of RAR as a valuable clinical tool.

The predictive value of RAR in assessing the prognosis of HF patients with pneumonia may stem from its ability to integrate multi-dimensional pathophysiological information, including inflammation, oxidative stress, nutritional status, and organ function, thereby reflecting the extent of systemic dysfunction in these patients. Elevated RDW, is strongly linked to chronic inflammation and oxidative stress. Pro-inflammatory cytokines (e.g., IL-6, TNF-α) and reactive oxygen species disrupt erythropoiesis, leading to the release of immature RBC and increased RDW (12). In HF patients with pneumonia, pulmonary infection exacerbates systemic inflammation via pathogen-associated molecular patterns and cytokine storms, further driving RDW elevation (13, 14). Simultaneously, as a key free radical scavenger, albumin depletion contributes to endothelial dysfunction and myocardial injury, further compromising cardiovascular integrity (15, 16). Thus, RAR encapsulates a self-perpetuating cycle of inflammation and oxidative damage, predisposing patients to multiorgan failure and increased mortality risk. Additionally, nutritional status is a crucial determinant of RAR. As a hallmark of malnutrition and hypercatabolism, hypoalbuminemia is highly prevalent in advanced HF and critically ill patients. HF-associated gut congestion and anorexia reduce protein intake, while concurrent infectious states elevate metabolic demands, accelerating albumin catabolism (17, 18). Malnutrition further impairs immune function and tissue repair, increasing susceptibility to refractory infections and delayed recovery. Moreover, micronutrient deficiencies including iron and vitamin B12 further contribute to ineffective erythropoiesis, worsening RDW variability and reducing oxygen transport capacity (19, 20). This crosstalk highlights RAR as a surrogate biomarker of global physiological reserve depletion, providing a more comprehensive assessment of patient status compared to traditional biomarkers such as natriuretic peptide and C-reactive protein.

The application of machine learning in our study, particularly the LightGBM model, enhanced the predictive accuracy for in-hospital mortality. The model demonstrated stable discrimination across both internal and external validation cohorts, underscoring the robustness and generalizability of our findings. SHAP analysis provided invaluable interpretability, allowing us to visualize the contribution of each feature to mortality risk at both the population and individual levels. By calculating the SHAP values, we can break down the contribution of individual features (21), such as RAR, renal function indices, and the use of RAASi or beta blockers. At the population level, SHAP revealed that RAR, along with RAASi use, lymphocyte percentage is one of the most influential factors in predicting mortality risk. These findings are clinically meaningful, as they underscore how inflammatory and nutritional status—reflected by RAR—play pivotal roles in the prognosis of HF patients with pneumonia. On the individual level, SHAP provided detailed insights into how specific features influence the mortality risk of individual patients. For instance, in certain patients, higher RAR was associated with a substantially increased risk of mortality, while the presence of RAASi or beta blockers provided a protective effect, shifting the risk toward a lower prediction. This level of interpretability not only improves transparency but also empowers clinicians to make more informed decisions based on individualized patient data, moving beyond a one-size-fits-all model. These insights complement traditional regression models by identifying nonlinear relationships and interactions that may otherwise be overlooked. For instance, while linear models suggest the direct contribution of individual predictors, SHAP uncovers complex interactions—such as between inflammatory markers and treatment regimens—that influence patient outcomes in a more nuanced manner. This enhanced understanding of the model's decision-making process is consistent with the growing body of literature that integrates SHAP into clinical applications (22, 23).

Our study also highlights the clinical utility of the developed machine learning model. We created a web-based calculator derived from the final LightGBM model, enabling real-time, individualized estimation of in-hospital mortality risk. This platform allows clinicians to input key patient data, such as demographic information, comorbidities, and laboratory values, to obtain personalized mortality risk predictions. The accessibility and ease of use of the tool—without the need for specialized programming skills—make it a practical addition to clinical workflows.

The real clinical impact of this tool lies in its potential to facilitate bedside decision-making. For instance, clinicians could use the web-based calculator to rapidly assess the mortality risk of a patient with HF and pneumonia, helping them determine the most appropriate level of care and interventions. The interpretability provided by SHAP values further enhances this decision-making process, as clinicians can understand why a particular patient is classified as high or low risk. However, while the tool shows promise, we acknowledge that prospective usability testing and multicenter validation are needed to fully assess its clinical effectiveness and potential impact on patient outcomes.

### 4.1 Limitations of the study

Despite these findings, several limitations must be considered when interpreting the results. First, the retrospective study design inherently precludes causal inference, and residual confounding from unmeasured factors, such as socioeconomic status and treatment adherence, cannot be fully excluded. Second, although external validation helps mitigate center-specific bias, the single-center recruitment strategy and limited ethnic diversity of the cohort may restrict the generalizability of the findings. Third, the lack of phenotypic stratification for HF and pneumonia limits insights into potential heterogeneity in RAR's prognostic utility across disease subtypes. Future work requires prospective multicenter validation with subtype stratification, coupled with mechanistic trials testing whether RAR-directed interventions (e.g., anti-inflammatory therapies, albumin supplementation) improve survival beyond reflecting disease severity. Additionally, the absence of biomarkers like NT-proBNP, CRP, and LVEF is a limitation due to their high missing proportions in the dataset, leading to their exclusion from the model. This highlights the advantage of RAR as an easily accessible and cost-effective biomarker, especially in settings where these specialized markers are unavailable. Finally, a potential limitation is overfitting in the machine learning model. Although the training set showed a high AUC (0.939), the AUCs for the internal (0.735) and external (0.733) validation sets were lower. However, the close similarity between the internal and external validation AUCs suggests reasonable model generalizability. We applied regularization and early stopping to mitigate overfitting, but further refinement is needed to improve model robustness across different datasets.

## 5 Conclusions

In conclusion, this study highlights the utility of RAR as a prognostic biomarker and its successful integration into machine learning models for predicting 31-day in-hospital mortality in HF patients with pneumonia. By combining traditional biomarkers with machine learning approaches, this research underscores the potential for improved risk stratification, enhanced model interpretability, and personalized clinical decision-making, paving the way for precision medicine in high-risk patient management.

## Data Availability

The raw data supporting the conclusions of this article will be made available by the authors, without undue reservation.
