# Supplementary material for: Red blood cell distribution width to albumin ratio predicts mortality in heart failure patients with pneumonia
Source: Front Cardiovasc Med. 2026 Feb 5;13:1638901. doi: 10.3389/fcvm.2026.1638901 (PMC12916672; doi:10.3389/fcvm.2026.1638901)
Supplement: Supplementary file 1 [file Datasheet1.pdf]

Supplementary Table S1. ICD Codes for heart failure and pneumonia.

	**ICD Code**
**Heart Failure**	39891, 40201, 40211, 40291, 40401, 40403, 40411, 40413, 40491, 40493, 4280, 42820, 42821, 42822, 42823, 42830, 42831, 42832, 42833, 42840, 42841, 42842, 42843, 4289, I0981, I110, I130, I132, I502, I5020, I5021, I5022, I5023, I503, I5030, I5031, I5033, I504, I5040, I5041, I5042, I5043, I508, I5082, I5083, I5084, I5089, I509, I9713, I97130, I97131, O29122, O29129, P290, 4281, I5081, I50810, I50811, I50812, I50813, I50814, I50, O2912, O29121, O29123
**Pneumonia**	01160, 01161, 01162, 01163, 01164, 01165, 01166, 0521, 0551, 0730, 11505, 11515, 11595, 1304, 3201, 4800, 4801, 4802, 4803, 4808, 4809, 481, 4820, 4821, 4822, 48230, 48231, 48232, 48239, 48240, 48241, 48242, 48249, 48281, 48282, 48283, 48284, 48289, 4829, 4830, 4831, 4838, 4841, 4843, 4845, 4846, 4847, 4848, 485, 486, 4870, 48801, 48811, 48881, 4957, 4958, 4959, 5060, 5070, 5071, 5078, 51630, 51632, 51633, 51635, 51636, 51637, 5171, 7700, 99731, 99732, A0103, A3701, A3711, A3781, A3791, A5004, A5484, B012, B052, B0681, B250, B7781, J09X1, J100, J1000, J1008, J110, J1100, J1108, J12, J120, J121, J122, J123, J128, J1281, J1282, J1289, J129, J13, J14, J15, J150, J151, J152, J1520, J1521, J15211, J15212, J1529, J153, J154, J155, J156, J157, J158, J159, J16, J160, J168, J17, J18, J180, J181, J182, J188, J189, J200, J67, J678, J679, J680, J69, J690, J691, J698, J8281, J8282, J8411, J84111, J84113, J84114, J84116, J84117, J842, J851, J954, J95851, O2901, O29011, O29012, O29013, O29019, O740, O8901, P23, P230, P231, P232, P233, P234, P235, P236, P238, P239, U071

*Both the MIMIC-IV cohort and the First Affiliated Hospital of Zhengzhou University cohort include various types of pneumonia. However, due to the overlapping data collection period with the COVID-19 pandemic, the proportion of COVID-19 is relatively higher in the First Affiliated Hospital of Zhengzhou University cohort.

Supplementary Table S2. Proportion of missing data and variable handling.

Variable	Missing (n)	Missing (%)	Variable handling
RAR	0	0	
**Demographics**			
Gender	0	0	
Age	0	0	
BMI	1926	41.7	Excluded
**Comorbidities**			
Myocardial infarction	0	0	
Diabetes	0	0	
Hypertension	0	0	
Hepatopathy	0	0	
Atrial fibrillation	0	0	
Kidney disease	0	0	
Malignant tumor	0	0	
Chronic pulmonary disease	0	0	
Rheumatic disease	0	0	
Cerebrovascular disease	0	0	
**Disease Characteristics**			
Diagnosis Sequence	0	0	
Ischemic HF	0	0	
**Medications**			
Glucocorticoid	0	0	
Antibiotics	0	0	
Anticoagulant	0	0	
Beta blocker	0	0	
RAASi	0	0	
Diuretic	0	0	
Antiplatelet drugs	0	0	
NSAIDs	0	0	
SGLT2i	0	0	
ARNI	0	0	
**First laboratory results upon ICU admission**			
RBC	0	0	
Plt	5	0.1	
WBC	4	0.1	
Hb	2	0	
Neut	431	9.3	
Neut abs	1830	39.6	Excluded
Lymp	431	9.3	
Lymp abs	1796	38.9	Excluded
Monocyte	1830	39.6	Excluded
ALT	182	3.9	

AST	114	2.5	
ALP	119	2.6	
Cr	0	0	
BUN	1	0	
INR	87	1.9	
PT	84	1.8	
PTT	111	2.4	
K^+	4	0.1	
Na^+	0	0	
Ca^{2+}	0	0	
Cl^-	0	0	
P	1	0	
$HCO3^-$	1	0	
Glu	4	0.1	
CK	1365	29.6	Excluded
CKMB	1380	29.9	Excluded
NTproBNP	2375	51.4	Excluded
TnT	1658	35.9	Excluded
CRP	4240	91.8	Excluded
**Hemodynamic and Clinical Monitoring Parameters**			
CVP	3056	66.2	Excluded
LVEF average	3053	66.1	Excluded
Fluid Balance	2342	50.7	Excluded
**Vital signs**			
RR	0	0	
HR	0	0	
SBP	2149	46.5	Excluded
DBP	2149	46.5	Excluded
SpO2	0	0	

Supplementary Table S3. Comparison of variable distributions before and after multiple imputation.

Variable	Pre-imputation	Post-imputation
RAR	5.50 ± 1.67	5.50 ± 1.67
**Demographics**		
Male, n (%)	2660 (57.6%)	2660 (57.6%)
Age, years	72.27 ± 13.34	72.27 ± 13.34
**Comorbidities, n (%)**		
Myocardial infarction	1449 (31.4%)	1449 (31.4%)
Diabetes	2006 (43.4%)	2006 (43.4%)
Hypertension	3622 (78.4%)	3622 (78.4%)
Hepatopathy	593 (12.8%)	593 (12.8%)
Atrial fibrillation	1721 (37.3%)	1721 (37.3%)
Kidney disease	2004 (43.4%)	2004 (43.4%)
Malignant tumor	631 (13.7%)	631 (13.7%)
Chronic pulmonary disease	1965 (42.6%)	1965 (42.6%)
Rheumatic disease	221 (4.8%)	221 (4.8%)
Cerebrovascular disease	731 (15.8%)	731 (15.8%)
**Disease Characteristics, n (%)**		
Diagnosis Sequence	1577 (34.1%)	1577 (34.1%)
Ischemic HF	2585 (56.0%)	2585 (56.0%)
**Medications, n (%)**		
Glucocorticoid	2120 (45.9%)	2120 (45.9%)
Antibiotics	4560 (98.7%)	4560 (98.7%)
Anticoagulant	3975 (86.1%)	3975 (86.1%)
Beta blocker	3590 (77.7%)	3590 (77.7%)
RAASi	1693 (36.7%)	1693 (36.7%)
Diuretic	4061 (87.9%)	4061 (87.9%)
Antiplatelet drug	3012 (65.2%)	3012 (65.2%)
NSAIDs	3032 (65.7%)	3032 (65.7%)
SGLT2i	20 (0.4%)	20 (0.4%)
ARNI	57 (1.2%)	57 (1.2%)
**First laboratory results upon ICU admission**		
RBC, m/uL	3.50 ± 0.80	3.50 ± 0.80
Plt, K/uL	217.60 ± 117.36	217.51 ± 117.38
WBC, K/uL	13.44 ± 10.36	13.43 ± 10.36
Hb, g/dL	10.26 ± 2.28	10.26 ± 2.28
Neut, %	78.66 ± 13.65	78.64 ± 13.77
Lymp, %	11.18 ± 10.25	11.29 ± 10.44
ALT, IU/L	104.43 ± 439.14	102.05 ± 431.02
AST, IU/L	150.28 ± 618.01	151.63 ± 647.62
ALP, IU/L	115.87 ± 108.75	115.64 ± 108.02
Cr, mg/dL	1.92 ± 1.78	1.92 ± 1.78

BUN, mg/dL	38.56 ± 27.01	38.57 ± 27.02
INR	1.75 ± 1.26	1.75 ± 1.26
PT, sec	18.97 ± 12.88	18.98 ± 12.85
PTT, sec	41.76 ± 27.29	41.80 ± 27.39
K^{+}, mEq/L	4.33 ± 0.82	4.33 ± 0.82
Na^{+}, mEq/L	137.92 ± 6.03	137.92 ± 6.03
Ca^{2+}, mg/dL	8.39 ± 0.91	8.39 ± 0.91
Cl^{-}, mEq/L	101.50 ± 7.31	101.50 ± 7.31
P, mg/dL	4.07 ± 1.52	4.07 ± 1.52
$HCO3^{-}$, mEq/L	23.74 ± 5.91	23.73 ± 5.91
Glu, mg/dL	160.78 ± 89.73	160.79 ± 89.73
**Vital signs**		
RR, insp/min	21.33 ± 6.62	21.33 ± 6.62
HR, bpm	91.82 ± 31.62	91.82 ± 31.62
SpO2, %	96.04 ± 18.38	96.04 ± 18.38

Supplementary Table S4. Baseline Characteristics Before and After Propensity Score Matching.

Variable	Before PSM				After PSM			
	Low-RAR (n = 1574)	High-RAR (n = 1584)	*P*	SMD	Low-RAR (n = 998)	High-RAR (n = 998)	*P*	SMD
**Demographics**								
Male, n (%)	911 (57.9%)	932 (58.8%)	0.609	0.019	567 (56.8%)	577 (57.8%)	0.684	0.02
Age, years	72.93 (12.71)	73.51 (12.69)	0.194	0.046	73.58 (12.27)	73.26 (12.88)	0.569	0.025
**Comorbidities, n (%)**								
Myocardial infarction	496 (31.5%)	466 (29.4%)	0.215	0.045	319 (32.0%)	309 (31.0%)	0.664	0.022
Diabetes	639 (40.6%)	720 (45.5%)	0.007	0.098	438 (43.9%)	439 (44.0%)	1.000	0.002
Hypertension	1285 (81.6%)	1220 (77.0%)	0.002	0.114	794 (79.6%)	786 (78.8%)	0.700	0.02
Hepatopathy	153 (9.7%)	231 (14.6%)	<0.001	0.149	117 (11.7%)	128 (12.8%)	0.495	0.034
Atrial fibrillation	543 (34.5%)	640 (40.4%)	0.001	0.122	378 (37.9%)	373 (37.4%)	0.853	0.01
Kidney disease	613 (38.9%)	721 (45.5%)	<0.001	0.133	436 (43.7%)	430 (43.1%)	0.821	0.012
Malignant tumor	140 (8.9%)	256 (16.2%)	<0.001	0.221	120 (12.0%)	124 (12.4%)	0.838	0.012
Chronic pulmonary disease	692 (44.0%)	685 (43.2%)	0.71	0.015	445 (44.6%)	434 (43.5%)	0.652	0.022
Cerebrovascular disease	270 (17.2%)	248 (15.7%)	0.277	0.04	167 (16.7%)	159 (15.9%)	0.672	0.022
**Disease Characteristics, n (%)**								
Diagnosis Sequence	551 (35.0%)	510 (32.2%)	0.102	0.06	342 (34.3%)	332 (33.3%)	0.67	0.021
ischemic HF	886 (56.3%)	862 (54.4%)	0.307	0.038	555 (55.6%)	544 (54.5%)	0.653	0.022
**Medications, n (%)**								
Glucocorticoid	667 (42.4%)	741 (46.8%)	0.014	0.089	426 (42.7%)	469 (47.0%)	0.059	0.087
Anticoagulant	1268 (80.6%)	1411 (89.1%)	<0.001	0.239	813 (81.5%)	887 (88.9%)	<0.001	0.21
Beta blocker	1269 (80.6%)	1218 (76.9%)	0.012	0.091	809 (81.1%)	766 (76.8%)	0.021	0.106
RAASi	702 (44.6%)	474 (29.9%)	<0.001	0.307	562 (56.3%)	664 (66.5%)	<0.001	0.211
Diuretic	1425 (90.5%)	1399 (88.3%)	0.05	0.072	913 (91.5%)	883 (88.5%)	0.031	0.1
Antiplatelet drug	1120 (71.2%)	975 (61.6%)	<0.001	0.204	700 (70.1%)	632 (63.3%)	0.001	0.145
NSAIDs	1117 (71.0%)	984 (62.1%)	<0.001	0.188	697 (69.8%)	642 (64.3%)	0.01	0.117
**First laboratory results upon ICU admission**								

RBC, m/uL	3.67 ± 0.74	3.36 ± 0.78	<0.001	0.398	3.47 ± 0.71	3.46 ± 0.78	0.713	0.016
Plt, K/uL	212.62 ± 89.78	213.79 ± 105.31	0.738	0.012	212.06 ± 94.05	209.88 ± 102.55	0.621	0.022
WBC, K/uL	12.32 ± 5.85	12.87 ± 7.25	0.02	0.083	12.31 ± 6.02	12.27 ± 6.76	0.91	0.005
Hb, g/dL	10.96 ± 2.21	9.65 ± 1.98	<0.001	0.627	10.19 ± 1.98	10.12 ± 2.00	0.474	0.032
Neut, %	79.49 ± 10.08	79.91 ± 10.87	0.261	0.04	79.99 ± 9.93	79.73 ± 10.61	0.576	0.025
Lymp, %	11.43 ± 7.46	9.92 ± 7.30	<0.001	0.204	10.73 ± 7.12	10.60 ± 7.59	0.683	0.018
ALT, IU/L	52.03 ± 109.25	55.72 ± 123.47	0.373	0.032	52.75 ± 113.87	51.24 ± 111.17	0.765	0.013
AST, IU/L	73.14 ± 139.90	80.00 ± 176.38	0.226	0.043	72.28 ± 145.36	72.38 ± 148.17	0.988	0.001
ALP, IU/L	92.78 ± 48.05	114.45 ± 71.20	<0.001	0.357	98.36 ± 53.68	100.67 ± 56.68	0.351	0.042
Cr, mg/dL	1.59 ± 1.17	1.76 ± 1.22	<0.001	0.141	1.67 ± 1.22	1.70 ± 1.21	0.691	0.018
BUN, mg/dL	32.18 ± 20.32	38.55 ± 23.20	<0.001	0.292	35.00 ± 21.95	35.04 ± 21.21	0.974	0.001
INR	1.52 ± 0.70	1.65 ± 0.75	<0.001	0.185	1.58 ± 0.75	1.59 ± 0.68	0.794	0.012
PT, sec	16.55 ± 7.19	17.95 ± 7.75	<0.001	0.188	17.14 ± 7.73	17.23 ± 7.06	0.792	0.012
PTT, sec	35.86 ± 15.41	36.48 ± 14.22	0.238	0.042	36.06 ± 15.48	6.07 ± 14.33	0.99	0.001
K^+, mEq/L	4.29 ± 0.70	4.25 ± 0.72	0.176	0.048	4.28 ± 0.70	4.26 ± 0.74	0.5	0.03
Na^+, mEq/L	138.08 ± 4.88	138.31 ± 5.45	0.217	0.044	138.18 ± 5.05	138.24 ± 5.20	0.78	0.013
Ca^{2+}, mg/dL	8.55 ± 0.70	8.27 ± 0.75	<0.001	0.395	8.43 ± 0.69	8.38 ± 0.75	0.139	0.066
Cl^-, mEq/L	101.61 ± 6.11	102.10 ± 6.77	0.034	0.076	101.82 ± 6.31	101.86 ± 6.63	0.874	0.007
P, mg/dL	3.80 ± 1.18	3.93 ± 1.28	0.003	0.105	3.87 ± 1.23	3.85 ± 1.25	0.762	0.014
$HCO3^-$, mEq/L	24.25 ± 5.40	24.23 ± 5.58	0.885	0.005	24.42 ± 5.51	24.33 ± 5.44	0.725	0.016
Glu, mg/dL	157.76 ± 65.29	147.87 ± 65.19	<0.001	0.152	152.86 ± 59.81	151.40 ± 67.50	0.608	0.023
**Vital signs**								
RR, insp/min	20.72 ± 5.95	21.31 ± 6.14	0.006	0.097	20.90 ± 6.01	20.93 ± 5.87	0.919	0.005
HR, bpm	89.71 ± 25.24	92.97 ± 26.20	<0.001	0.127	90.80 ± 24.55	91.89 ± 26.15	0.340	0.043
SpO2, %	95.74 ± 4.51	95.81 ± 4.62	0.636	0.017	95.87 ± 4.32	95.83 ± 4.60	0.845	0.009

Supplementary Table S5. Baseline characteristics of the training and internal validation cohorts.

Variable	Training cohort (n = 2211)	Internal validation cohort (n = 947)	*P*
RAR	5.33 ± 1.43	5.35 ± 1.38	0.75
**Demographics**			
Male, n (%)	1290 (58.3%)	553 (58.4%)	1. 00
Age, years	73.26 ± 12.57	73.13 ± 13.00	0.80
**Comorbidities, n (%)**			
Myocardial infarction	658 (29.8%)	304 (32.1%)	0.21
Diabetes	935 (42.3%)	424 (44.8%)	0.21
Hypertension	1746 (79.0%)	759 (80.1%)	0.48
Hepatopathy	265 (12.0%)	119 (12.6%)	0.69
Atrial fibrillation	812 (36.7%)	371 (39.2%)	0.21
Kidney disease	943 (42.7%)	391 (41.3%)	0.50
Malignant tumor	291 (13.2%)	105 (11.1%)	0.12
Chronic pulmonary disease	957 (43.3%)	420 (44.4%)	0.61
Cerebrovascular disease	364 (16.5%)	154 (16.3%)	0.93
**Disease Characteristics, n (%)**			
Diagnosis Sequence	748 (33.8%)	313 (33.1%)	0.70
ischemic HF	1204 (54.5%)	544 (57.4%)	0.13
**Medications, n (%)**			
Glucocorticoid	995 (45.0%)	413 (43.6%)	0.50
Anticoagulant	1871 (84.6%)	808 (85.3%)	0.65
Beta blocker	1715 (77.6%)	772 (81.5%)	0.02
RAASi	799 (36.1%)	377 (39.8%)	0.06
Diuretic	1973 (89.2%)	851 (89.9%)	0.64
Antiplatelet drug	1455 (65.8%)	640 (67.6%)	0.36
NSAIDs	1464 (66.2%)	637 (67.3%)	0.60
**First laboratory results upon ICU admission**			
RBC, m/uL	3.51 ± 0.78	3.52 ± 0.76	0.70
Plt, K/uL	213.07 ± 98.31	213.52 ± 96.88	0.91
WBC, K/uL	12.47 ± 6.53	12.90 ± 6.74	0.09
Hb, g/dL	10.27 ± 2.21	10.36 ± 2.16	0.30
Neut, %	79.63 ± 10.38	79.85 ± 10.73	0.59
Lymp, %	10.73 ± 7.37	10.53 ± 7.52	0.49
ALT, IU/L	52.24 ± 110.93	57.71 ± 128.85	0.23
AST, IU/L	73.72 ± 148.80	83.26 ± 181.25	0.12
ALP, IU/L	102.97 ± 60.90	105.24 ± 63.62	0.34
Cr, mg/dL	1.67 ± 1.19	1.69 ± 1.21	0.66
BUN, mg/dL	34.92 ± 21.68	36.44 ± 22.83	0.08
INR	1.59 ± 0.73	1.58 ± 0.72	0.76

PT, sec	17.28 ± 7.57	17.19 ± 7.36	0.75
PTT, sec	36.28 ± 14.55	35.92 ± 15.47	0.54
K^+, mEq/L	4.26 ± 0.71	4.30 ± 0.72	0.18
Na^+, mEq/L	138.17 ± 5.14	138.27 ± 5.24	0.63
Ca^{2+}, mg/dL	8.42 ± 0.74	8.38 ± 0.74	0.19
Cl^-, mEq/L	101.79 ± 6.47	102.01 ± 6.42	0.37
P, mg/dL	3.86 ± 1.21	3.87 ± 1.28	0.71
$HCO3^-$, mEq/L	24.30 ± 5.49	24.10 ± 5.48	0.36
Glu, mg/dL	152.11 ± 65.57	154.41 ± 65.07	0.37
**Vital signs**			
RR, insp/min	21.02 ± 6.08	21.02 ± 5.99	1.00
HR, bpm	91.02 ± 25.34	92.10 ± 26.75	0.28
SpO2, %	95.81 ± 4.61	95.69 ± 4.45	0.49

Supplementary Table S6. Baseline characteristics of MIMIC-IV cohort and the external validation cohort.

**Variable**	**MIMIC-IV (n = 3,158)**	**External cohort (n = 1,110)**	***P***	***SMD***
RAR	5.34 ± 1.41	4.49 ± 1.17	<0.001	0.659
Age, years	73.22 ± 12.70	68.72 ± 14.18	<0.001	0.335
Hepatopathy	384 (12.2%)	299 (26.9%)	<0.001	0.379
Beta blocker	2487 (78.8%)	558 (50.3%)	<0.001	0.624
RAASi	1176 (37.2%)	337 (30.4%)	<0.001	0.146
NSAIDs	2101 (66.5%)	640 (57.7%)	<0.001	0.184
Plt, K/uL	213.21 ± 97.87	186.20 ± 89.53	<0.001	0.288
Lymp, %	10.67 ± 7.42	14.49 ± 10.47	<0.001	0.421
ALP, IU/L	76.58 ± 159.26	50.64 ± 104.93	<0.001	0.192
Cr, mg/dL	1.67 ± 1.20	1.91 ± 2.17	<0.001	0.135
BUN, mg/dL	35.38 ± 22.04	28.79 ± 20.33	<0.001	0.311
Na^+, mEq/L	138.20 ± 5.17	138.60 ± 5.29	0.025	0.078
P, mg/dL	3.86 ± 1.23	1.08 ± 0.39	<0.001	3.048

Supplementary Table S7. Pairwise DeLong tests for AUC comparison in the external validation cohort.

Comparator model	AUC	ΔAUC (LightGBM - Comparator)	Z	*P* (DeLong)	*P* (Holm-adjusted)
Logistic Regression	0.699	0.034	2.835	0.005	0.087
Decision Tree	0.624	0.109	6.314	<0.001	<0.001
Random Forest	0.712	0.021	1.695	0.090	1.000
KNN	0.664	0.069	4.232	<0.001	0.001
SVM	0.671	0.062	3.682	<0.001	0.005
Neural Network	0.689	0.044	2.927	0.003	0.072
XGBoost	0.673	0.061	4.562	<0.001	<0.001

Supplementary Table S8. External validation performance and calibration of the eight prediction models.

Model	Brier score	HL groups	HL P value	Calibration intercept	Calibration Slope
LightGBM	0.18	10	<0.001	0.51	0.826
Random Forest	0.175	10	<0.001	0.79	1.461
Logistic Regression	0.192	10	<0.001	0.456	0.727
Neural Network	0.177	10	<0.001	-0.014	0.772
XGBoost	0.197	10	<0.001	-0.205	0.359
SVM	0.19	10	<0.001	-0.166	0.605
KNN	0.183	10	<0.001	-0.747	0.154
Decision Tree	0.19	10	<0.001	0.336	0.874

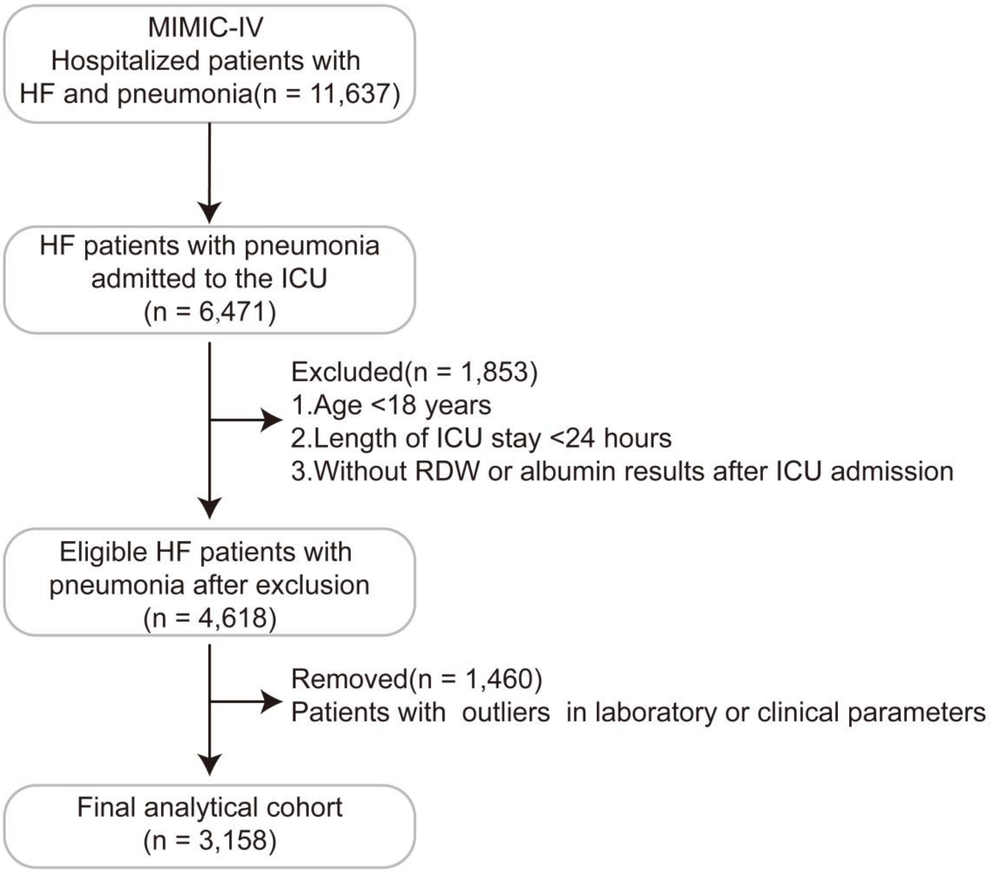

Supplementary Figure S1. Inclusion and exclusion criteria.

Variable	Count	Percent	HR (95% CI)	P-value	P for interaction
Overall	3158	100	1.36 (1.14-1.61)	<0.001	
Age					0.866
<60	473	15	1.42 (0.83-2.43)	0.2	
>=60	2685	85	1.34 (1.12-1.61)	0.001	
Gender					0.949
Female	1315	41.6	1.34 (1.02-1.76)	0.033	
Male	1843	58.4	1.37 (1.10-1.70)	0.006	
Diagnosis Sequence					0.987
0	2097	66.4	1.36 (1.11-1.67)	0.003	
1	1061	33.6	1.32 (0.97-1.81)	0.079	
Ischemic HF					0.059
No	1410	44.6	1.11 (0.85-1.45)	0.437	
Yes	1748	55.4	1.58 (1.26-1.98)	<0.001	
Myocardial infarction					0.091
No	2196	69.5	1.22 (0.99-1.50)	0.057	
Yes	962	30.5	1.71 (1.26-2.32)	0.001	
Diabetes					0.076
No	1799	57	1.20 (0.97-1.49)	0.095	
Yes	1359	43	1.68 (1.27-2.23)	<0.001	
Hypertension					0.255
No	653	20.7	1.66 (1.14-2.40)	0.008	
Yes	2505	79.3	1.27 (1.05-1.55)	0.014	
Hepatopathy					0.913
No	2774	87.8	1.34 (1.11-1.61)	0.002	
Yes	384	12.2	1.39 (0.89-2.16)	0.143	
Atrial fibrillation					0.886
No	1975	62.5	1.34 (1.07-1.66)	0.009	
Yes	1183	37.5	1.38 (1.05-1.82)	0.02	
Kidney disease					0.967
No	1824	57.8	1.37 (1.09-1.72)	0.008	
Yes	1334	42.2	1.32 (1.02-1.70)	0.033	
Malignant tumor					0.533
No	2762	87.5	1.31 (1.09-1.57)	0.004	
Yes	396	12.5	1.57 (0.93-2.65)	0.088	
Chronic pulmonary disease					0.726
No	1781	56.4	1.39 (1.11-1.74)	0.004	
Yes	1377	43.6	1.31 (1.01-1.71)	0.042	
Cerebrovascular disease					0.571
No	2640	83.6	1.39 (1.14-1.69)	0.001	
Yes	518	16.4	1.25 (0.87-1.80)	0.23	
Glucocorticoid					0.396
No	1750	55.4	1.27 (1.01-1.61)	0.043	
Yes	1408	44.6	1.45 (1.12-1.86)	0.004	
Anticoagulant					0.996
No	479	15.2	1.46 (0.93-2.28)	0.099	
Yes	2679	84.8	1.41 (1.17-1.70)	<0.001	
Beta blocker					0.503
No	671	21.2	1.49 (1.11-1.99)	0.008	
Yes	2487	78.8	1.23 (0.99-1.51)	0.058	
RAASi					0.211
No	1982	62.8	1.29 (1.06-1.56)	0.009	
Yes	1176	37.2	0.84 (0.56-1.28)	0.421	
Diuretic					0.599
No	334	10.6	1.65 (1.03-2.64)	0.037	
Yes	2824	89.4	1.29 (1.07-1.55)	0.007	
Antiplatelet drug					0.223
No	1063	33.7	1.16 (0.89-1.52)	0.281	
Yes	2095	66.3	1.41 (1.13-1.76)	0.002	
NSAIDs					0.08
No	1057	33.5	1.09 (0.83-1.42)	0.536	
Yes	2101	66.5	1.45 (1.16-1.81)	0.001	

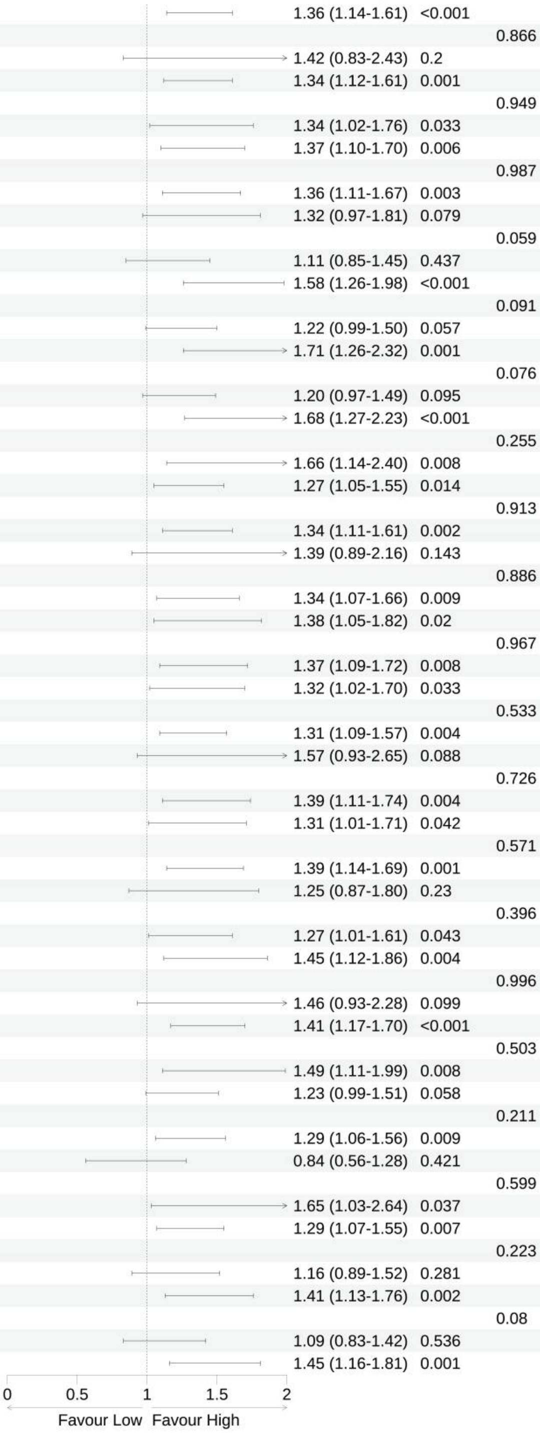

Supplementary Figure S2. Subgroup analysis for the association between RAR and 31-day in-hospital mortality in heart failure patients with pneumonia. Diagnosis Sequence = 1: Heart failure diagnosis precedes pneumonia; Diagnosis Sequence = 0: Pneumonia diagnosis precedes heart failure.

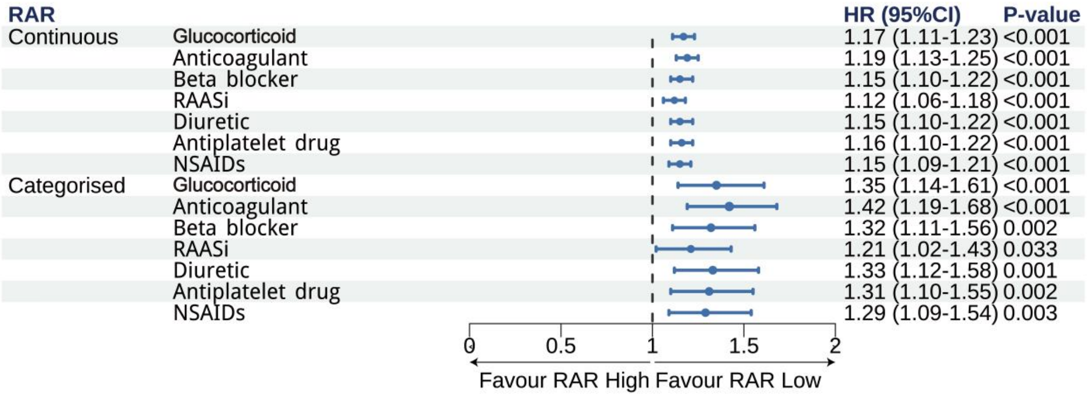

Supplementary Figure S3. Sensitivity analysis of RAR in drug-adjusted models for heart failure patients with pneumonia.

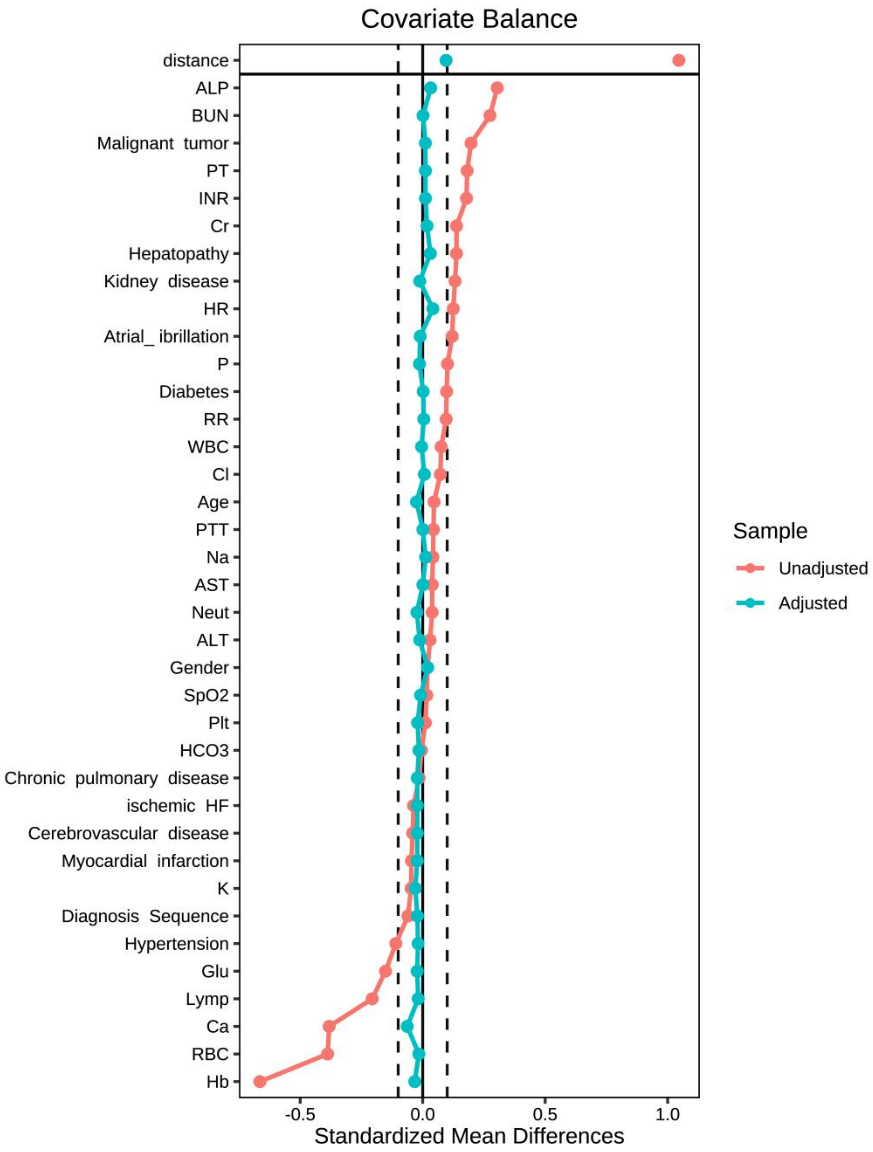

Supplementary Figure S4. Love Plot for Covariate Balance Before and After Propensity Score Matching.

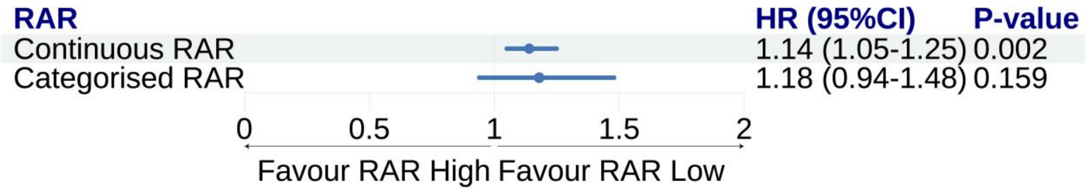

Supplementary Figure S5. Cox regression analysis assessing the association between RAR and in-hospital mortality after propensity score matching.

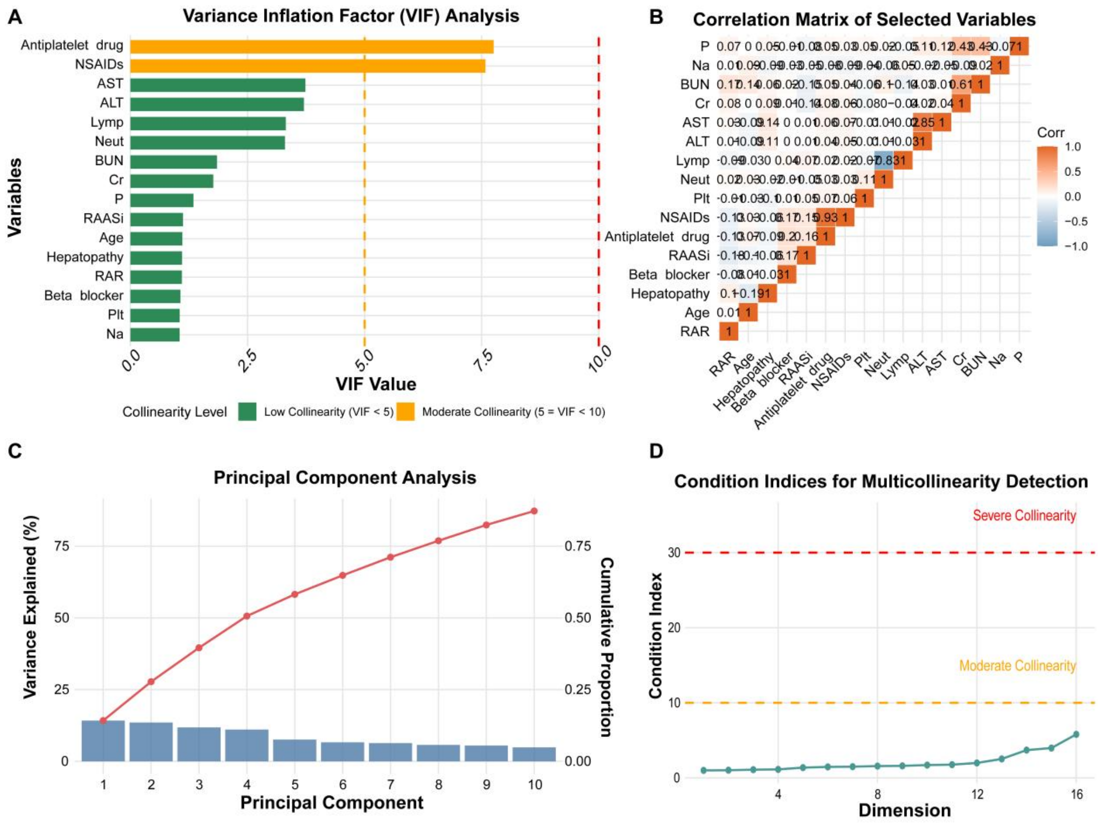

Supplementary Figure S6. Comprehensive multicollinearity diagnostics of candidate predictors. (A) Variance inflation factor (VIF) analysis of selected variables. Bars indicate VIF values for each predictor; the dashed vertical lines denote common thresholds. (B) Pearson correlation matrix of selected variables, with color intensity reflecting correlation strength. (C) Principal component analysis (PCA) showing the proportion of variance explained by each principal component (bars) and the cumulative explained variance (line). (D) Condition indices across dimensions for multicollinearity detection; dashed horizontal lines indicate thresholds for moderate (condition index = 10) and severe (condition index = 30) collinearity.
